# Systematic Construction and Validation of a Novel Ferroptosis-Related Gene Model for Predicting Prognosis in Cervical Cancer

**DOI:** 10.1155/2022/2148215

**Published:** 2022-07-28

**Authors:** Wentao Qin, Can He, Daqiong Jiang, Yang Gao, Yu Chen, Min Su, Yuanjun Yang, Zhao Yang, Hongbing Cai, Hua Wang

**Affiliations:** ^1^Department of Gynecological Oncology, Zhongnan Hospital of Wuhan University, Hubei Clinical Cancer Study Center, Hubei Key Laboratory of Tumor Biological Behaviors, Wuhan, China; ^2^Department of Obstetrics and Gynecology, Xiangyang No. 1 People's Hospital, Jinzhou Medical University Union Training Base, China

## Abstract

**Methods:**

Datasets containing RNA sequencing and corresponding clinical data of cervical cancer patients were obtained from searching publicly accessible databases. The “NMF” R package was conducted to calculate the matrix of the screened prognosis gene expression. Ferroptosis-related differential genes in cervical cancer were detected using the “limma” R function and WGCNA. The least absolute shrinkage and selection operator (LASSO) algorithm and Cox regression analysis were conducted to develop a novel prognostic signature. The prediction model was verified by the nomogram integrating clinical characteristics; the GSE44001 dataset was used as an external verification. Then, the immune status and tumor mutation load were explored. Finally, immunohistochemistry as well as quantitative polymerase chain reaction (RT-qPCR) was utilized to ascertain the expression of FRGs.

**Results:**

Two molecular subgroups (cluster 1 and cluster 2) with different FRG expression patterns were recognized. A ferroptosis-related model based on 4 genes (VEGFA, CA9, DERL3, and RNF130) was developed through TCGA database to identify the unfavorable prognosis cases. Patients in cluster 1 showed significantly decreased overall survival in contrast with those in cluster 2 (*P* < 0.05). The LASSO technique and Cox regression analysis were both utilized to establish the independence of the prognostic model. The validity of nomogram prognostic predictions has been well demonstrated for 3- and 5-year survival in both internal and external data validation cohorts. These two subgroups showed striking differences in tumor-infiltrating leukocytes and tumor mutation burden. The low-risk subgroup showed a longer overall survival time with a higher immune cell score and higher tumor mutation rate. Gene functional enrichment analyses revealed predominant enrichment in various tumor-associated signaling pathways. Finally, the expression of each gene was confirmed by immunohistochemistry and RT-qPCR.

**Conclusion:**

A novel and comprehensive ferroptosis-related gene model was proposed for cervical cancer which was capable of distinguishing the patients independently with high risk for poor survival, and targeting ferroptosis may represent a promising approach for the treatment of CC.

## 1. Introduction

Cervical cancer (CC) represents the third major contributor to cancer-related fatality in women worldwide [1, 2]. Radical hysterectomy and radiotherapy with or without chemotherapy led to a favorable prognosis in early-stage CC patients. However, follow-up data have shown that recurrent or metastatic cervical cancer remains refractory with only 17% of a 5-year survival rate after the above treatment [3]. Platinum-based first-line chemotherapy and anti-PD-L1 immunotherapy showed a less than satisfactory effect [4, 5]. It will be helpful to develop a novel prognostic model considering we lack predictive biomarkers for cervical cancer and the limited treatment strategies available.

Accumulating data suggest that ferroptosis is crucial for the development and treatment of resistance in a variety of malignancies [6–9]. It is currently considered that the functional mechanism of ferroptosis is related to four processes, including intracellular iron metabolism level, lipid peroxide content, and glutathione peroxidase [10]. Chemotherapy, radiotherapy, molecular targeted therapy, immunotherapy, traditional Chinese medicine treatment, and nanotechnology treatment can result in tumor ferroptosis in different ways [11, 12]. Tumor therapies targeting ferroptosis contribute to overcoming the resistance of existing therapies and can restore the sensitivity of tumor cells to therapeutic drugs. For example, Song and coworkers found that using nanoparticle-induced ferroptosis and blockade of programmed death ligand 1 (PD-L1) at the same time efficiently restrains the size of melanoma cells and lung metastasis of breast cancers, implying the application prospect of ferroptosis combined with immune checkpoint inhibitors [13]. In addition, Yao and his colleagues found induction of ferroptosis by anti-LCN2 inhibits the growth of liver cancer [14]. Also, Tso and coworkers found that drugs inducing ferroptosis displayed an orthogonal therapeutic approach which increased the differentiation plasticity of melanoma and improved the efficacy of antitumor treatment [15]. Therefore, ferroptosis-based therapy shows important prospects in combination therapy of cancers [16].

Hence, we created a prognostic model premised on the FRGs [17–19] and verified its prognosis-predictive efficacy in CC. Then, we studied the relationship of the two subgroups in the model with immune status in CC. This research is aimed at presenting the role of ferroptosis in the prognosis of patients with CC and offering novel insight into the targeted treatment of CC.

## 2. Materials and Methods

### 2.1. Data Collection and Processing

The RNA-seq data and the related clinical data of CC and normal specimens were retrieved from TCGA database, GTEx database, and GSE44001 dataset in the GEO database; somatic mutation datasets of tumor samples were acquired from TCGA database. In particular, TCGA database included the RNA-seq data as well as related clinical data of 306 CC patients and 3 normal specimens, the GTEx database included the RNA-seq data of 78 normal samples, and the GEO dataset included RNA-seq and clinical data of 300 tumor specimens. 60 ferroptosis-related genes constructed by the risk model were retrieved from the previous research [20–22].

### 2.2. Identification of Molecular Subtypes of FRGs in Cervical Cancer and Differentially Expressed Genes (DEGs)

The FRGs obtained in TCGA dataset were incorporated into the univariate model, and the prognosis-related genes were screened according to the following cutoff value: *P* < 0.01. The “NMF” R function was utilized to derive and unsupervised the matrix of the screened prognosis gene expression. The molecular subtypes of ferroptosis-related genes in cervical cancer were identified utilizing cluster analysis. The relevant parameters were selected as follows, the method adopts “brunet,” the number of iterations (nrun) is adjusted to 10, and the ranks are set to 2 to 10.

The differential expression genes with different ferroptosis genotyping were identified with the “limma” R function with the threshold values of FDR < 0.05, log_2_ | fold change (FC) | ≥2. The coexpression network between genes and clinical characteristics was generated utilizing the “WGCNA” R program [23], and significant models were identified. The subtype-related hub genes of the significant modules were detected utilizing the “VennDiagram” R program.

### 2.3. Creation and Verification of a Prognostic Model Premised on Differential Molecular Subtype-Related Genes

The dataset of cervical cancer in TCGA database was categorized into the training and testing sets in a 7 : 3 ratio. To search for subtype-related hub genes having prognostic values in the training set, the univariate Cox regression analysis was executed (*P* < 0.05). To determine which genes had the most predictive significance, a LASSO regression analysis using the R program “glmnet” was done on the genes listed above, and a prognostic model was generated. Then, to determine the risk prediction model, a multivariate Cox regression analysis was undertaken, and the regression coefficients of every gene included in the model were determined. Below is the equation utilized to generate each patient's risk score in the training set: riskScore = ∑^1^_*n*_Coef(mRNAn) × Expr(mRNAn). The median value was taken as the standard for classifying patients into high- and low-risk groups. The Kaplan-Meir (K-M) survival curve and ROC curve were plotted using “Survival” and “timeROC” R packages, respectively, to determine the stability of the model. Next, the “Regplot” R package was used to incorporate clinical features (including age, grade, stage, and BMI) into the model and construct a nomogram. The nomogram was verified for stability with the ROC curve and calibration curve. Test sets were utilized to internally validate the performance of the risk model. The GSE44001 dataset then was utilized for external verification.

### 2.4. Consensus Clustering Analysis of Ferroptosis-Related Differential Expressed Genes (DEGs) between Different Molecular Subtypes

Ferroptosis-related DEGs between different molecular subtypes were obtained by the “limma” R package, filtered by the cutoff values of discovery rate (FDR < 0.05) and ∣logFC | ≥1. Consensus clustering analysis of ferroptosis-related DEGs was performed with the “ConsensusClusterPlus” package. The proportion parameter of resampled samples (pItem) was 80%, the maximum evaluated category number (maxK) was 9, and the clustering distance was selected as “Euclidean.” The optimal number of clusters was selected as a *k* value, and different subgroups were divided according to the *k* value. Besides, for dimensionality reduction analysis between these subgroups, principal component analysis (PCA) by the “Boruta” package was conducted.

### 2.5. Functional Annotation of GO and KEGG

The functional enrichment analysis of Gene Ontology (GO) and Kyoto Encyclopedia of Genes and Genomes (KEGG) was undertaken to investigate fundamental mechanisms. Adjusted *P* < 0.05 denoted a significant difference.

### 2.6. Analysis of the Association of Risk Score for Cervical Cancer with Immune Infiltration

When comparing high- and low-risk groups, we conducted a single-sample GSEA (ssGSEA) using the “GSVA” R function to determine the relative infiltration levels of 28 distinct types of immune cells in the tumor microenvironment (TME). ESTIMATE algorithm via the “estimate” function was utilized to evaluate the TME in the high- and low-risk subgroups from three aspects of tumor purity, stromal score, and immune score.

### 2.7. Collection of Somatic Alteration Data

To determine the tumor mutation burden (TMB) in various ferroptosis types of CC, nonsynonymous mutations were calculated. The somatic alteration data of each tumor sample was downloaded from TCGA database. After that, we identified driver genes through the R “maftool” program according to the subtypes and compared the somatic alterations. We used the top 20 mutation frequency as a proxy for the total mutation rate.

### 2.8. Analysis of Quantitative Reverse Transcription-Polymerase Chain Reaction (qRT-PCR)

Both cervical cancer and adjacent noncancerous tissues used in this study were obtained from postoperative patients with cervical cancer from 2021 to 2022 in Zhongnan Hospital of Wuhan University. All samples were obtained through review by the ethics committee, and the patients' informed consent was acquired. We extracted RNA from specimens by utilizing the TRIzol reagent (Invitrogen, USA), followed by reverse transcription into cDNA utilizing the QuantiTect Reverse Transcription Kit (Qiagen, Germany). Quantitative PCR (qPCR) is a technique for measuring the amount of DNA present in a sample in real time. With the aid of SYBR-Green (Takara, Japan), real-time qPCR assays were carried out, and expression levels were standardized to GAPDH levels.  Table 1 consists of a collection of primer sequences utilized in this study.

### 2.9. Immunohistochemical Validation

After fixing cervical cancer specimens in 10% formalin followed by embedding in paraffin, samples were processed into 5 *μ*m thicknesses sequential segments. To suppress endogenous peroxidase activities, tissue segments were deparaffinized using ethanol and subsequently blocked. The next step involved heating the samples in a boiler to collect antigens and subsequent cooling to ambient temperature and blocking with goat serum at 37°C for 30 minutes. After that, the specimens were subjected to overnight incubation at 4°C with rabbit anti-VEGFA (bs-20393R), anti-CA9 (bs-4029R), anti-DERL3 (bs-14281R), and anti-RNF130 (bs-9252R) (Beijing Boaosen Biotechnology Co., Ltd., China). They were again incubated for 30 minutes with horseradish peroxidase-coupled goat anti-rabbit secondary antibody (bs-40295G-HRP, Beijing Boaosen Biotechnology Co., Ltd., China) at 37° C. In the next step, the specimens were, respectively, stained with 3,3′-diaminobenzidine (DAB). After that, the specimens were subjected to dehydration, clearing in xylene, and mounting.

### 2.10. Statistical Analysis

R (version 3.6.2) was utilized to analyze all statistical data. The Kaplan-Meier technique was utilized to analyze the link between the prognoses of each eigenvalue. The survival curve was examined utilizing the log-rank test. Multivariate and univariate Cox and Cox regression analyses were carried out to find out independent prognostic factors and used LASSO regression for overfitting screening to convert continuous variables (such as age) into dichotomous variables. Methods: data analysis between the two subgroups was performed utilizing a two-tailed *t*-test, and Welch's *t*-test was used when necessary. The statistical significance of all analyses was determined at *P* < 0.05.

## 3. Results

### 3.1. Clustering of Molecular Subtypes of Cervical Cancer

**Figure 1:**
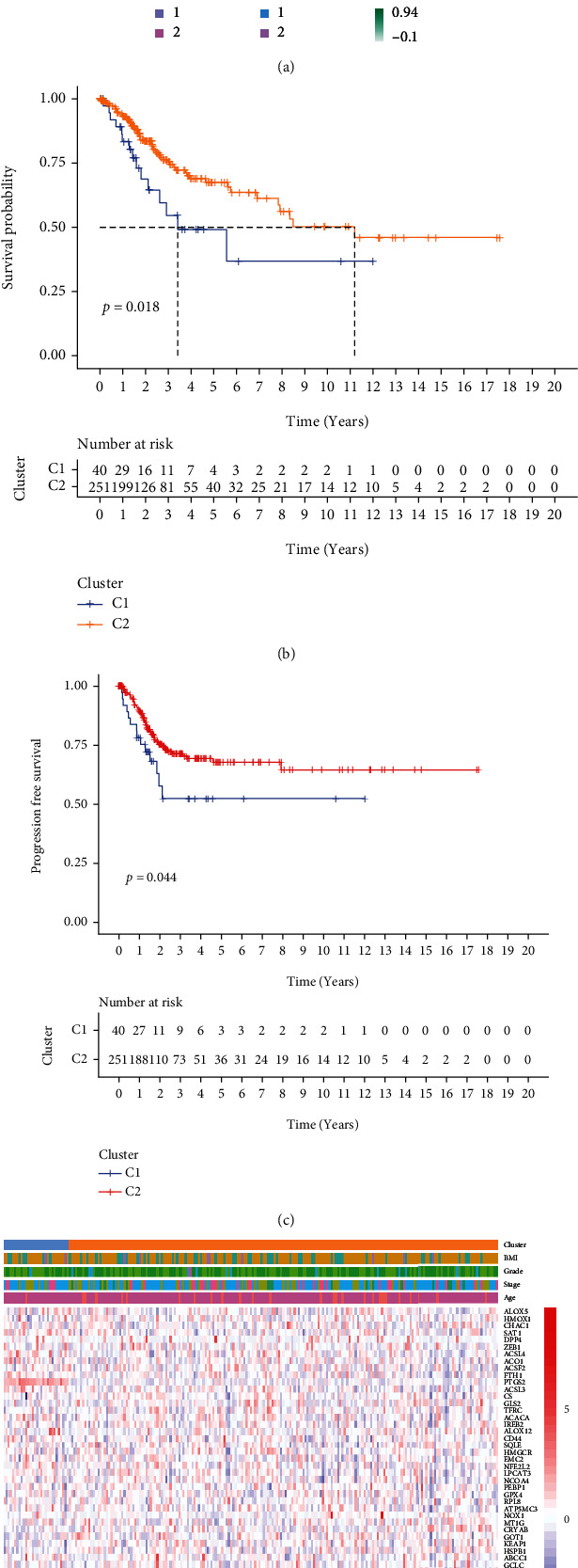
Identification of ferroptosis-associated subtypes using nonnegative matrix cluster analysis. (a) The optimal *k* value was determined to be 2, and TCGA cervical cancer samples could be divided into two subgroups, C1 and C2. (b, c) K-M survival curves of overall survival (OS) and disease-free survival (PFS) in C1 and C2 subgroups. (d) Heat map of age, stage, grade, BMI, and ferroptosis-related gene expression across subgroups.

291 CC patients were included based on TCGA database, who had been partitioned into C1 and C2 subtypes by the NMF cluster analysis of 60 FRGs (Figure 1(a)). The results of survival analysis suggested that the PFS and OS of patients with the C2 subtype were considerably higher as opposed to those of patients with C1 subtypes (Figures 1(b) and 1(c)). And the heat map demonstrated the differential expression of relevant clinical information between C1 and C2 subtypes, including age, BMI, tumor stage, and grade (Figure 1(d)).

### 3.2. Identification of DEGs in Cervical Cancer and Construction of Ferroptosis-Related Modules by WGCNA

**Figure 2:**
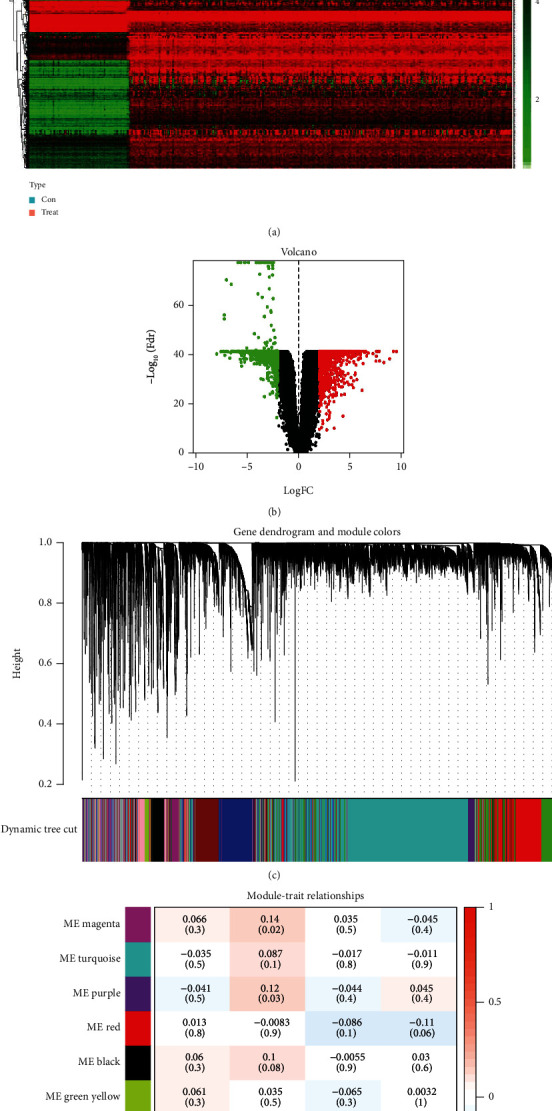
Identification of differential molecular subtype genes associated with ferroptosis. (a, b) mRNA-seq differential analysis of cervical cancer and normal tissue heat maps and volcano plots, with red for highly expressed genes and green for lowly expressed genes. (c) Based on the hierarchical clustering analysis of TCGA dataset, genes with similar characteristics are assigned to modules of the same color. (d) Heat map of correlations between eigenvalues and individual modules, red indicates positive correlation and blue indicates negative correlation.

In total, 2355 DEGs were detected between cervical cancer and adjacent nontumorous samples, including 1083 upmodulated and 1272 downmodulated genes (Figures 2(a) and 2(b)). Based on the soft-thresholding power equal to 9 (*β* = 9), we stratified the DEGs to obtain a hierarchical clustering tree by WGCNA analysis (Figure 2(c)). And a total of 11 coexpressed modules were detected according to similar characteristics of DEGs. The grey and blue modules were related to the ferroptosis-related phenotype in CC. The grey module illustrated the strongest correlation (*R* = 0.25, *P* < 0.001) (Figure 2(d)). To construct a prognostic model associated with ferroptosis, ∗∗ genes in the grey module were taken to intersect with 1789 genes in the blue module, and finally, 180 key genes were obtained.

### 3.3. Construction of a Prognostic Model Related to Ferroptosis

**Figure 3:**
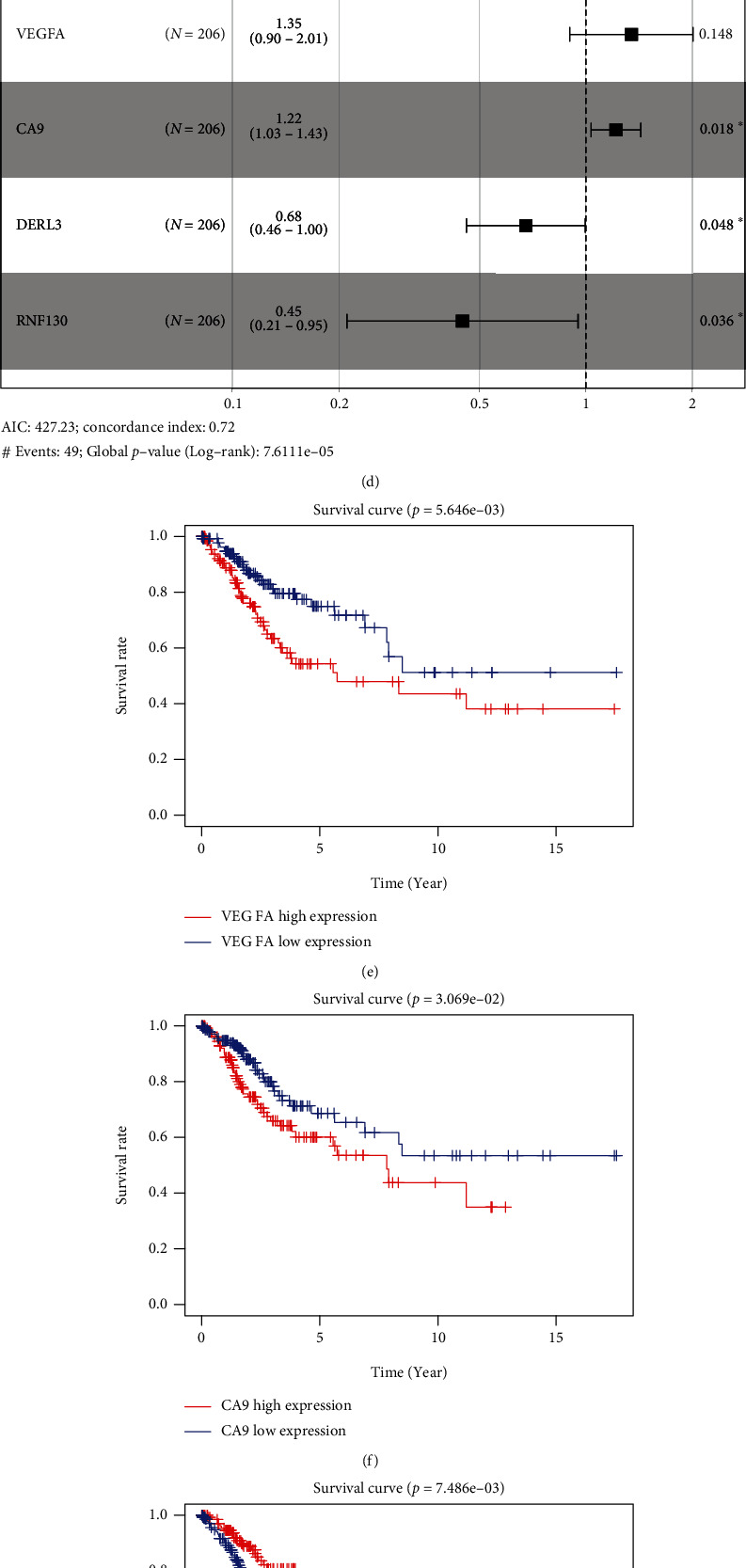
Screening of prognostic model factors. (a) Univariate Cox regression analysis screened out 4 prognostic related genes. (b) Trajectory changes of 4 genes. (c) Confidence interval for each *λ* value. (d) Multivariate Cox regression analysis. (e–h) K-M survival curves of 4 genes with independent prognostic potency.

**Figure 4:**
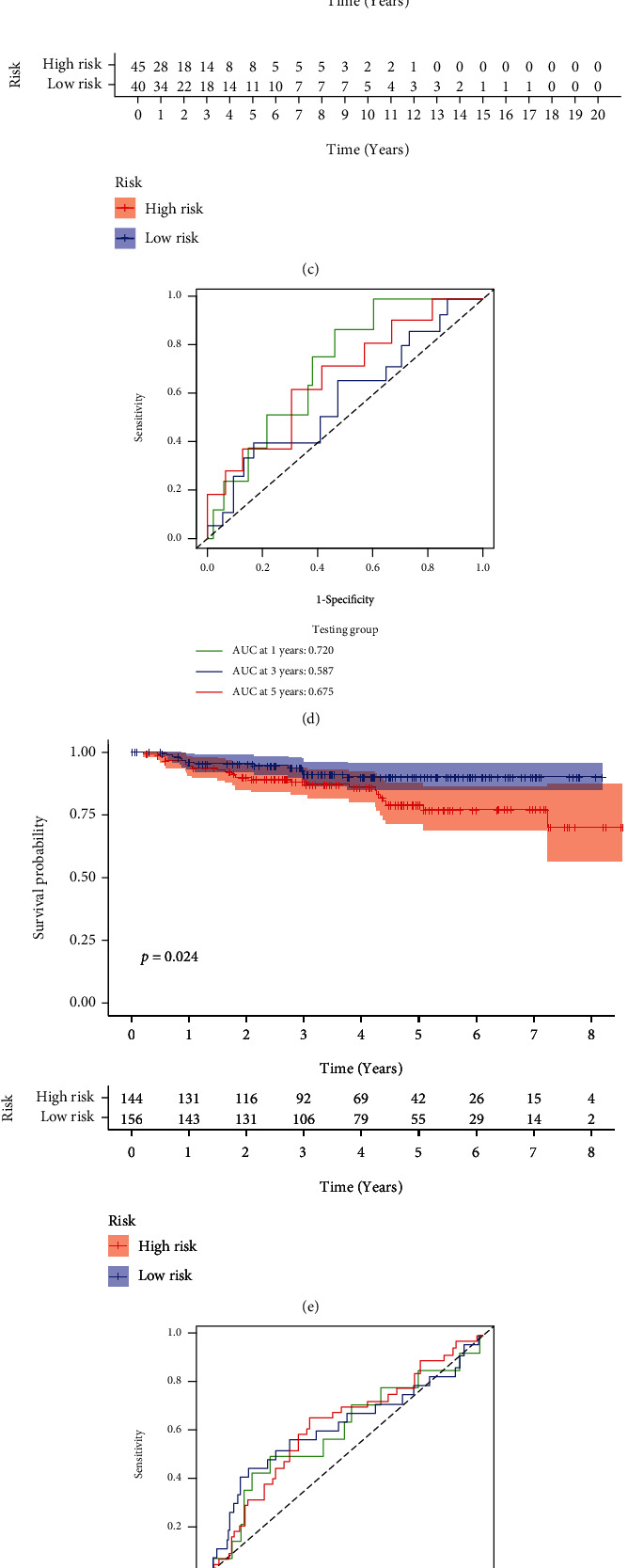
Construction and validation of ferroptosis typing-related gene signatures. (a, c, e, g) K-M survival curves of high- and low-risk groups in training set, validation set, GEO cohort, and TCGA total cohort. (b, d, f, h) ROC curves of 1, 3, and 5 years for training set, validation set, GEO cohort, and TCGA total cohort.

CC specimens in TCGA cohorts were randomized and classified to obtain 206 specimens in the training set and 85 specimens in the test set. Four genes were linked to the prognosis of ferroptosis in cervical cancer, namely, RNF130, CA9, DERL3, and VEGFA, which were obtained by univariate Cox regression analysis in the training set (Figure 3(a)). To eliminate the overfitting of this model, the training cohort was subjected to LASSO regression analysis and multivariate Cox regression analyses (Figures 3(b)–3(d)). And the K-M curves of the four genes were obtained (Figures 3(e)–3(h)). Finally, the prognostic model for cervical cancer was established based on above genes (riskScore = 0.297∗VEGFA + 0.196∗CA9 + (−0.391)∗DERL3 + (−0.804)∗RNF130). Based on the K-M curves for the training cohort, it was illustrated that patients in the high-risk group experienced unfavorable prognostic outcomes in contrast with the low-risk group (Figure 4(a)). Additionally, after plotting the receiver operating characteristic (ROC) curves, we found that the values of the area under the curve (AUC) were 0.737, 0.734, and 0.706 over 1, 3, and 5 years correspondingly (Figure 4(b)). The result showed that the ferroptosis-related prognostic model had good diagnostic efficacy in cervical cancer.

We carried out test cohort validation as well as external validation to additionally confirm the robustness of the model. In the test cohort and GEO database, a poor prognostic outcome remained strongly comparable to the findings from the training cohort (Figures 4(c) and 4(e)). The AUC at 1, 3, and 5 years was 0.720, 0.587, and 0.675 in the test cohort (Figure 4(d)) and 0.595, 0.613, and 0.613 in the GEO external validation cohort, correspondingly (Figure 4(f)). In addition, we also performed validation on the entire dataset of TCGA, which yielded highly similar results (Figure 4(g)). The AUC was 0.727, 0.704, and 0.706 over 1, 3, and 5 years correspondingly (Figure 4(h)). In both the internal and external validation cohorts, it was shown that the ferroptosis-related prognostic model exhibited good performance.

### 3.4. Analysis of the Relationship of Risk Score with Clinicopathological Parameters

**Figure 5:**
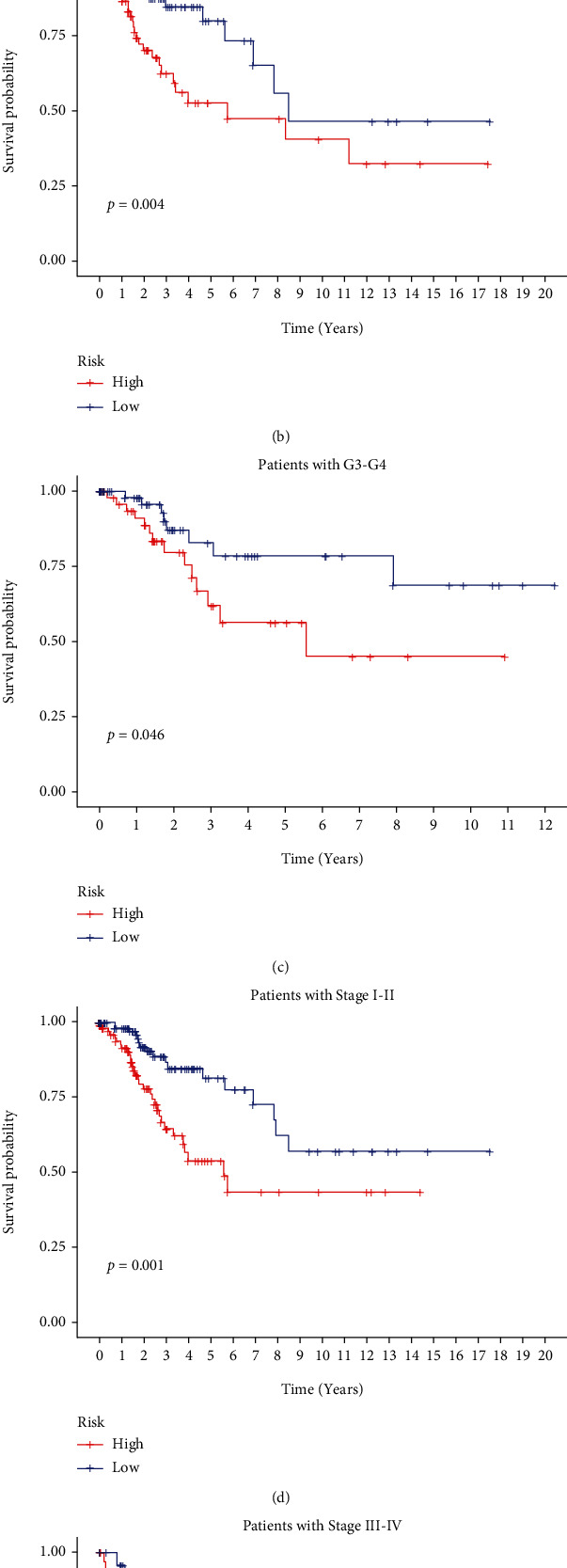
Survival analysis of clinically relevant factors. (a–e) Age (<65), grade (G1-G2, G2-G3), and stage (stage I-II, stage III-IV) K-M survival curves of high- and low-risk groups.

**Figure 6:**
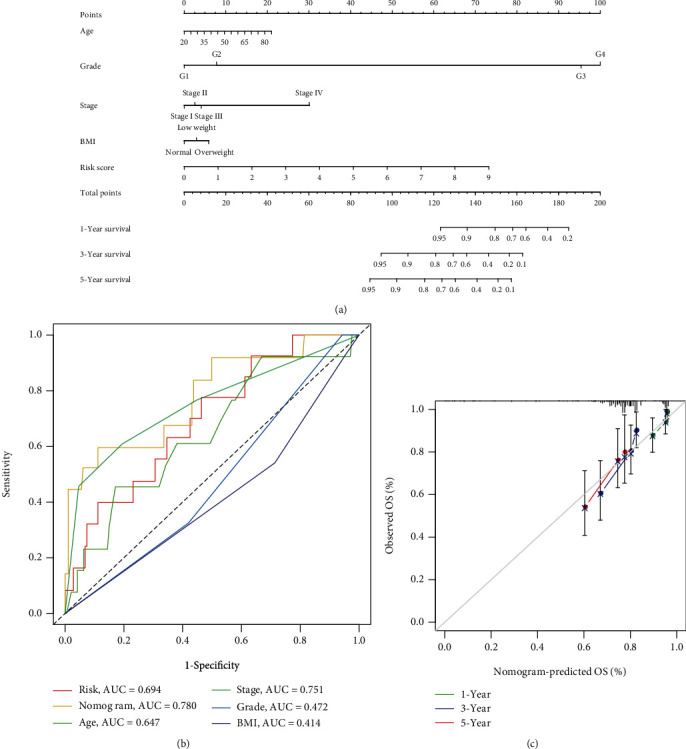
Construction and validation of a prognostic model for cervical cancer patients. (a) Nomogram for predicting cervical cancer patients at 1, 3, and 5 years based on TCGA total cohort. (b) ROC curve containing age, stage, grade, BMI, risk score, and nomogram. (c) Calibration curves for nomogram 1-, 3-, and 5-year predictive power tests.

In univariate and multivariate Cox regression analyses, the risk score and clinical characteristics, including, age, BMI, stage, and grade, were taken into consideration. The findings of a univariate Cox regression analysis illustrated that age, risk score, and tumor stage were all substantially linked to overall survival (OS) in CC and were all independent prognostic variables in this disease (Table 2). Survival analysis illustrated that the OS of CC patients with different ages, tumor grades, and stages were significantly different (Figure 5). We created a nomogram to anticipate the OS of CC patients based on clinical features and risk scores (Figure 6(a)). Additionally, the calibration curve and ROC curve were used to examine the nomogram's performance (Figures 6(b) and 6(c)). The findings illustrated that the model was capable of accurately predicting the OS of CC patients.

### 3.5. Consistent Cluster Analysis of Differential Genes of C1/C2 Subtypes of Cervical Cancer

**Figure 7:**
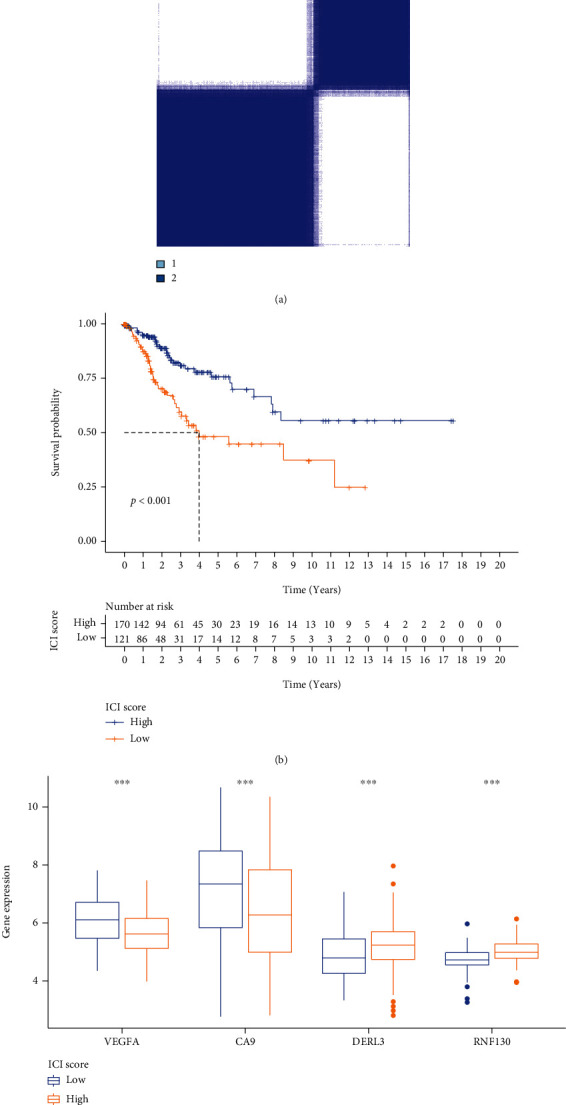
Consistent clustering analysis of C1 and C2 subtype differential genes. (a) Clustering results of differential genes between the two subtypes. (b) K-M survival curve of high and low PCA score groups of differential genes. (c) Differential expression box plots of the four modeled genes in the high and low PCA scoring groups. ^∗^*P* < 0.05; ^∗∗^*P* < 0.01; ^∗∗∗^*P* < 0.001.

The ferroptosis-related DEGs between C1 and C2 subtypes of cervical cancer were obtained by the “limma” R program. Unsupervised cluster analysis was conducted on the DEGs associated with ferroptosis, and *k* = 2 was chosen as the grouping optimal value (Figure 7(a)). PCA was applied to the different subgroups to compute each subgroup's risk score. Depending on their median risk scores, all patients were categorized into two groups: high- and low-risk groups. The K-M curves illustrated that the OS of patients in the low-risk group was considerably elevated in contrast with the high-risk group (Figure 7(b)). Additionally, the expression of four key genes had significant differences (Figure 7(c)).

### 3.6. Enrichment Analysis of Differential Genes by GO and KEGG

**Figure 8:**
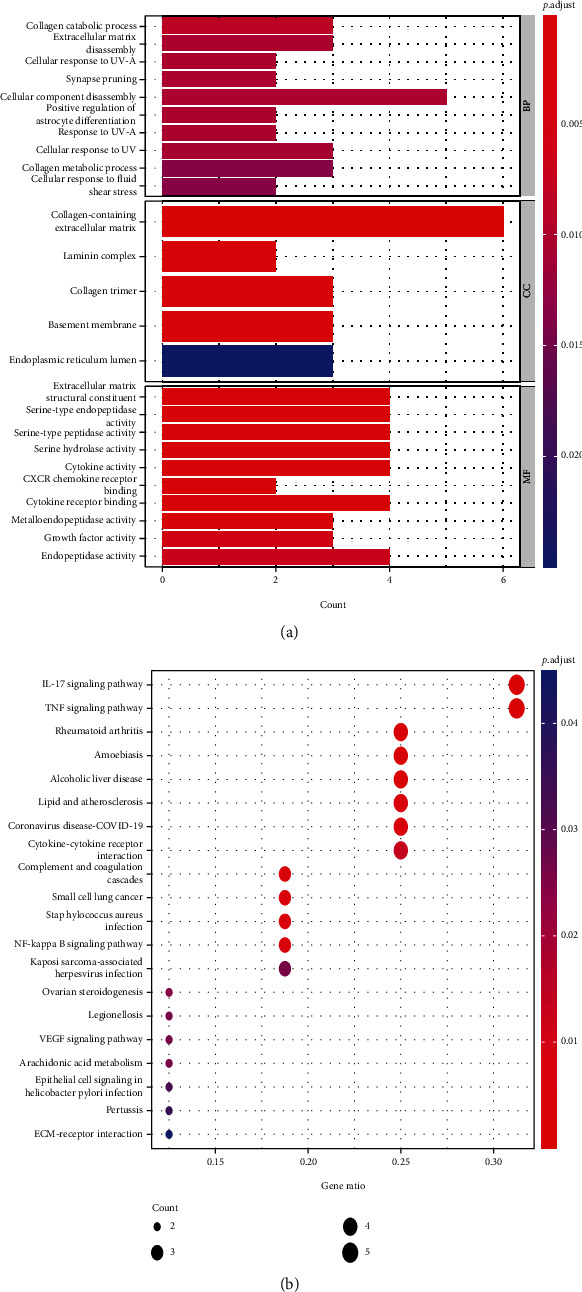
GO and KEGG analysis of differential genes. (a) Histogram of GO enrichment analysis of differential genes. (b) Bubble plot of KEGG enrichment analysis of differential genes.

We performed GO and KEGG enrichment analyses among the ferroptosis-related DEGs (Figures 8(a) and 8(b)). The GO enrichment results are mainly reflected in three aspects, including biological function (BP), cellular components (CC), and molecular functions (MF). In this work, BP was found to be relevant to the process of collagen decomposition, cell component decomposition, disassembly, extracellular matrix, and positive regulation of organisms. CC exhibited predominant enrichment in collagen-containing, extracellular matrix and laminin complex. A considerable enrichment of MF was found in the extracellular matrix, structural constituent, serine-type endopeptidase, CXCR chemokine receptor, etc. KEGG was shown to have predominant enrichment in NF-kappa B signaling pathway, TNF signaling pathway, L-17 signaling pathway, and VEGF signaling pathway.

### 3.7. Analysis of the Association of Risk Score of Cervical Cancer with Immune Infiltration

**Figure 9:**
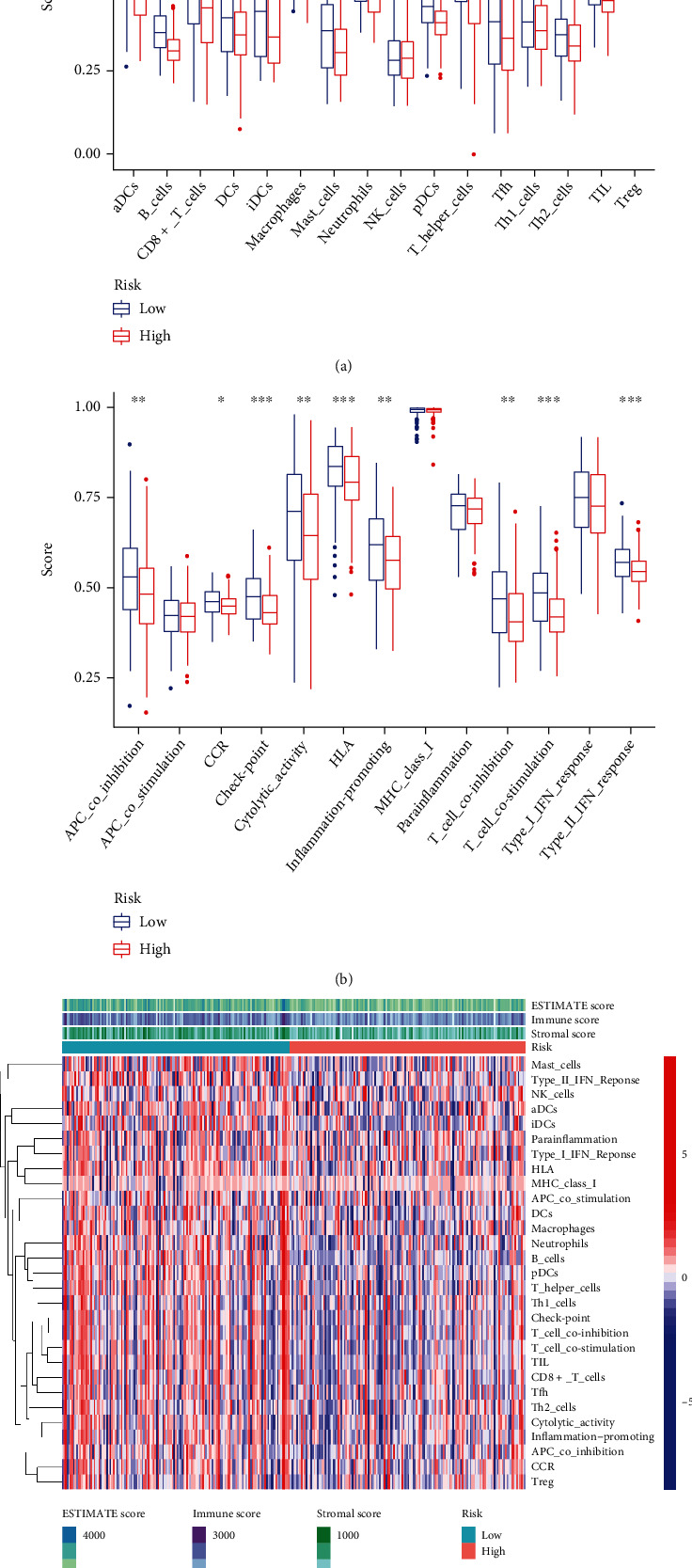
Immune infiltration correlation analysis. (a, b) Analyze the immune differences between high- and low-risk samples from 16 immune cells and 13 immune-related functions. (c) Heat map of stromal score, immune score, and estimate score in high- and low-risk groups. ^∗^*P* < 0.05; ^∗∗^*P* < 0.01; ^∗∗∗^*P* < 0.001.

In view of the negative link of risk score with cervical cancer prognosis, we performed GSEA between low- and high-risk groups of the C1 and C2 subtypes. 16 distinct types of immune cells and 13 types of immune-associated mechanisms in cervical cancer were obtained by the ssGSEA algorithm. The findings illustrated that the infiltration level of immune cells in the low-risk group was largely elevated in contrast with the high-risk group (Figures 9(a) and 9(b)). It suggested that the content of immune cells in cervical cancer might affect the patients' survival duration to some degree. By utilizing the ESTIMATE algorithm of the “estimate” R package, the heat map showed that the expression levels of stromal score, immune score, and estimate score in low-risk groups were substantially elevated as opposed to the high-risk group (*P* < 0.05) (Figure 9(c)).

### 3.8. Analysis of the Relationship of Ferroptosis-Related Phenotype with TMB in Cervical Cancer

**Figure 10:**
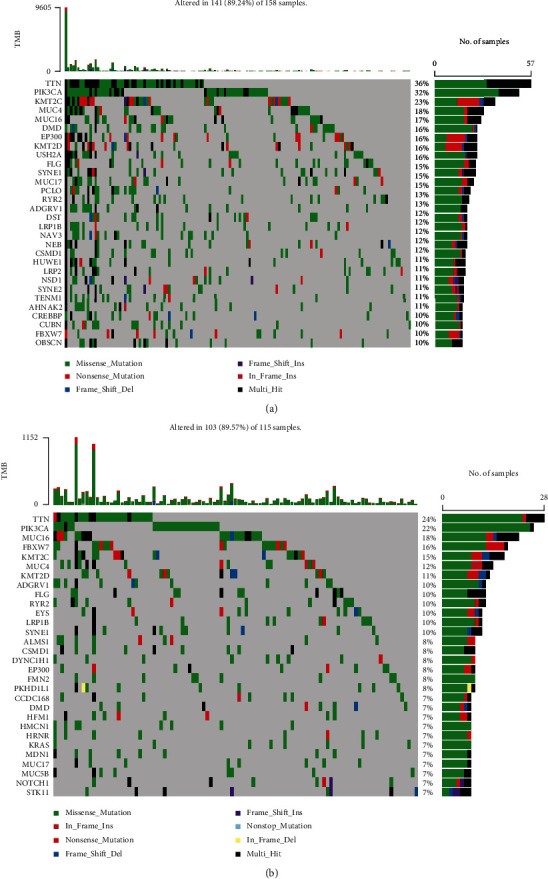
TMB. (a) Waterfall plot of somatic mutation status in high-risk samples. (b) Waterfall plot of somatic mutation status in a low-risk population.

The TMB was a way for somatic cells to increase the types of antigens by mutation and thus resist cancer. Numerous researches have suggested that TMB might be a potential biomarker that can determine the patients' responsiveness to the immune checkpoint blockers. We calculated the two risk groups' TMB by “maftools” packages separately (Figures 10(a) and 10(b)). Higher TMB could generate more mutations and be more conducive to the body's resistance to the development of cancer. In this study, The waterfall diagrams illustrated that TTN and PIK3CA genes in the low- and high-risk groups had the highest mutation rates. TDN accounted for 36% of mutations in the high-risk group and 24% of mutations in the low-risk group, whereas PIK3CA accounted for 32% of mutations in the high-risk group and 22% of mutations in the low-risk group.

### 3.9. Real-Time Quantitative Reverse Transcription PCR (qRT-PCR)

**Figure 11:**
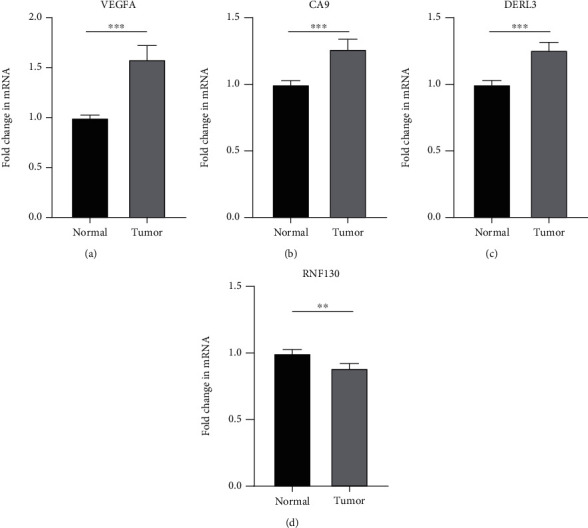
qRT-PCR. (a–d) The results showed that the expression of VEGFA, CA9, and DERL3 in cervical cancer was significantly higher than that in normal samples, while the expression of RNF130 in cervical cancer was lower than that in the normal group. ^∗^*P* < 0.05; ^∗∗^*P* < 0.01; ^∗∗∗^*P* < 0.001.

The findings obtained from qRT-PCR illustrated that the expression levels of VEGFA, CA9, and DERL3 in cervical cancer specimens were dramatically elevated in contrast with those in normal specimens, whereas the expression levels of RNF130 were lower contrasted to those in normal specimens (Figure 11).

### 3.10. Immunohistochemical (IHC) Experiments

**Figure 12:**
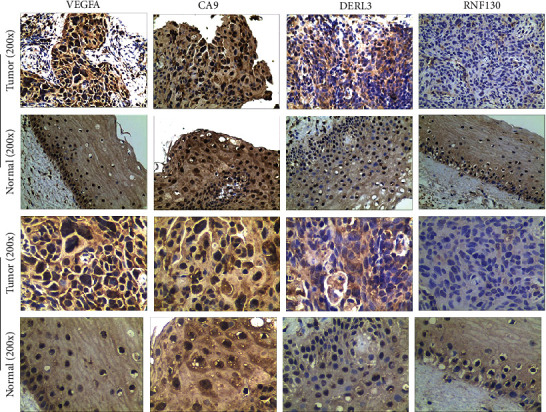
Protein expression of modeled genes in cervical cancer samples and normal samples. Shown in the figure are the results of immunohistochemistry under ×200 and ×400 magnification. The results showed that the positive expression of VEGFA, CA9, and DERL3 in cervical cancer was significantly higher than that in normal samples, while the expression of RNF130 in cervical cancer was lower than that in the normal group.

The IHC data revealed that the expression of VEGFA, CA9, and DERL3 in CC specimens was more obvious as opposed to that in normal samples, while the expression of RNF130 was decreased in tumor tissues (Figure 12).

## 4. Discussion

In the current study, the novel designed model with four genes (RNF130, CA9, DERL3, and VEGFA) yielded high specificity and sensitivity in identifying prognosis in the cervical cancer. Functional analyses revealed that immune-related pathways were enriched. Unlike previous studies only with selection operator (LASSO) algorithm and Cox regression analysis, we also adopted internal and external data in validation in order to screen specificity as possible for the creation of the prognostic signature. The most important thing is that we verified the results of our first step difference analysis through qRT-PCR and immunohistochemical studies, which is of great significance to our subsequent research and conclusions.

Firstly, in our study, we performed NMF cluster analysis on the genes of ferroptosis-related cervical cancer and defined two subtypes with different clinical characteristics in 291 cervical cancer cases of TCGA (Figure 1). Then, we used differential analysis and WGCNA analysis to screen the differentially expressed genes and select the intersection genes (Figure 2). The NMF-based strategy guaranteed the high homogeneity of clinical characteristics and diagnosis of patients with different molecular characteristics of cervical cancer ferroptosis, which reflect the characteristics of cervical cancer ferroptosis more accurately. Based on the above intersection genes, a novel prognostic model integrating 4 ferroptosis-related genes (RNF130, CA9, DERL3, and VEGFA) was firstly constructed (Figure 3). RNF130 is a protein that has E3 ubiquitin ligase activity and is implicated in the modulation of biological activities, including, apoptosis, gene transcription, intracellular signal transduction, and DNA repair. Additionally, the overexpression of RNF130 has been identified in numerous cancers while the opposite expression was reported in other cancers [24]. Carbonic anhydrase IX (CA9), which belongs to the carbonic anhydrase family and is associated with hypoxic cancer cells, performs an instrumental function in the balance between intracellular and extracellular pH through chemical reactions and further produces an acidic extracellular microenvironment [25, 26]. DERL3 is closely associated with the degradation of misfolded proteins in the endoplasmic reticulum, which is responsible for regulating epithelial-mesenchymal transition [27, 28]. VEGFA is well known for its role in promoting tumor angiogenesis [29, 30]. However, CA9, DERL3, and VEGFA genes were all upregulated while the opposite was reported for RNF130 in cervical cancer and was linked to unsatisfactory prognosis in the present research. It remains unclear whether the 4 genes affect the cervical cancer patients' prognoses by influencing the process of ferroptosis since there were few studies focusing on these genes.

Secondly, we used Cox regression analysis and LASSO algorithm to construct nomograms to predict the 1-, 3-, and 5-year survival rates of patients. In this constructed nomogram (Figure 6(a)), in terms of weighted scores, the ferroptosis-related gene signature had the greatest score, followed by clinical stage and grade. Three-year and five-year survival rates were used to illustrate the effectiveness of nomogram-based prognostic prediction, and these results were verified across both the internal and external data validation cohorts. In summary, this study uses a variety of methods to comprehensively verify the validity of the model and demonstrated the predictive effectiveness of the prognostic model.

Thirdly, though the mechanism of tumor susceptibility to iron intoxication has become a hot topic in the past few years [15], the probable modulatory mechanisms between tumor and iron intoxication remain uncertain. The GO and KEGG pathway enrichment analyses of the differential genes have shown that the differential genes were not only in cellular component (CC) but also in molecular function (MF), biological process (BP), and so on. All three are enriched and are also associated with various signaling-related pathways in cervical cancer (Figures 8(a) and 8(b)). Consequently, given the critical role of ferroptosis in tumorigenesis, we have improved our understanding of the mechanisms of the tumor progression and prognosis of cervical cancer.

Furthermore, the association between ferroptosis and immune cell infiltrating of cervical cancer is yet to be illustrated even though numerous research reports have focused on the process of ferroptosis in cancers in recent years [31, 32]. We discovered enhanced infiltration levels of CD8+ T cells in the low-risk group and so did the other 15 immune-associated cells as opposed to the high-risk group (Figures 9(a) and 9(b)). This directly demonstrated that immune cell infiltration was linked to a favorable prognosis in CC patients [33], consistent with numerous previous studies [34, 35]. Meanwhile, the activation of antitumor immune response including the activation of CD8+ T cells and IFN*α* cascade immune response represented lower risk scores. Therefore, improved antitumor immunity in patients with cervical cancer at low risk might explain the favorable prognosis.

Of note, tumor mutation burden (TMB) was typically considered the number of somatic nonsynonymous mutations per megabase pair (Mb) in a specific genomic region, and the number of tumor somatic mutations is positively correlated with immune and radiotherapy efficacy [36–38]. The results showed that TMB was enhanced in the high-risk group in contrast with the low-risk group (Figures 10(a) and 10(b)), and the two most frequently mutated genes were TTN and PIK3CA. A higher proportion of patients with TTN and PIK3CA somatic mutations was detected in the high-risk group in the cohort. We thought that variant TTN and PIK3CA was the driving factor of the tumorigenesis of cervical cancer. And contributed to increased cancer risk, we could use the mutation as a marker for predicting immune efficacy. In conclusion, ferroptosis of cervical cancer may be closely related to TMB, and further study was expected for its underlying mechanism in the future.

Finally, we carried out qRT-PCR and immunohistochemical studies on the four ferroptosis genes linked to the cervical cancer patients' prognoses. The findings illustrated that VEGFA, CA9, and DERL3 were highly expressed while the opposite was reported for RNF130 in CC (Figures 11 and 12). These results showed the accuracy of our first step differential analysis and improved the credibility of subsequent studies and also confirmed the association of risk genes with ferroptosis, further verifying the predictive power of our model.

Therefore, a novel and comprehensive ferroptosis-related gene model was proposed for cervical cancer that is capable of identifying patients independently at high risk for poor survival. We propose that targeting ferroptosis might be a promising approach for treating CC. To thoroughly comprehend the function performed by ferroptosis in the tumor or the TME and the mechanism through which it influences the cervical cancer patients' prognoses, further research is warranted.

## Figures and Tables

**Table 1:** Forward and reverse sequences of primers for VEGFA, CA9, DERL3, RNF130, and GAPDH.

Primer	Sequence (5′ to 3′)
VEGFA-F	ATCGAGTACATCTTCAAGCCAT
VEGFA-R	GTGAGGTTTGATCCGCATAATC
CA9-F	GAAGAAAACAGTGCCTATGAGC
CA9-R	TGTAGTCAGAGACCCCTCATAT
DERL3-F	CTCACTTTCCAGGCACCGTTCC
DERL3-R	GTAGTAGATATGGCCCACCGCAATC
RNF130-F	TCAACATTGCAGTAACAAGTGG
RNF130-R	TACATGTCCAAAGAAGGTTCGA
GAPDH-F	CAGGAGGCATTGCTGATGAT
GAPDH-R	GAAGGCTGGGGCTCATTT

**Table 2:** Univariate and multivariate Cox regression analysis of clinically relevant factors.

	Univariate Cox			Multifactor Cox		
	HR	95% CI	*P*	HR	95% CI	*P*
Age	2.81	1.48~5.33	<0.01	2.44	1.28~4.64	<0.01
Stage	1.73	1.32~2.26	<0.01	1.60	1.21~2.11	<0.01
Grade	1.00	0.61~1.66	0.99			
BMI	0.70	0.45~1.10	0.12			
riskScore	1.66	1.32~2.08	<0.01	1.53	1.21~1.92	<0.01

## Data Availability

The data used in this research come from open online databases, which included the GEO (https://www.ncbi.nlm.nih.gov/geo/) and TCGA (https://portal.gdc.cancer.gov/).
